# Comparison of First Trimester Screening for Down’s Syndrome Using Free Beta-Human Chorionic Gonadotropin and Pregnancy-Associated Plasma Protein-A Levels between Spontaneous and IVF Pregnancies at 12 Weeks of Gestation

**DOI:** 10.22074/ijfs.2019.5295

**Published:** 2019-04-27

**Authors:** Robabeh Taheripanah, Maryam Talayeh, Marzieh Zamaniyan, Donya Khosravi, Anahita Taheripanah

**Affiliations:** 1 Infertility and Reproductive Health Research Center (IRHRC), Shahid Beheshti University of Medical Sciences, Tehran, Iran; 2 Imam Hossein Hospital, Obstetrics and Gynecology Department, Shahid Beheshti University of Medical Sciences, Tehran, Iran; 3 Diabetes Research Center, Mazandaran University of Medical Sciences, Sari, Iran; 4 Infertility Center, Department of Obstetrics and Gynecology, Mazandaran University of Medical Sciences, Sari, Iran; 5 Department of Molecular and Cellular Sciences, Faculty of Advanced Sciences and Technology Pharmaceutical Sciences Branch, Islamic Azad University, Tehran, Iran

**Keywords:** Chorionic Gonadotropin, Down Syndrome, *In Vitro* Fertilization, Pregnancy, Pregnancy-Associated Plasma Protein-A

## Abstract

**Background:**

In some previous studies, it was shown that first trimester screening tests produce equivocal results in in vitro fertilization (IVF) pregnancies. The purpose of this study was to compare free beta-human chorionic gonadotro- pin (β-hCG) and pregnancy-associated plasma protein-A (PAPPA) levels between single normal and IVF pregnancies during 11 to 13 week (+ 6 day) of gestational age.

**Materials and Methods:**

In this observational cohort study, 300 consecutive single IVF pregnancies and 700 single normal pregnancies were enrolled at about 11-13 week + 6 day gestational age and levels of free β-hCG and PAPPA were compared between the groups.

**Results:**

The results demonstrated that PAPPA (P=0.026) was significantly lower and β-hCG (P=0.030) was signifi- cantly higher in IVF pregnancies. The other factors including nuchal translucency (NT) and crown-rump length (CRL) and demographic characteristics did not significantly differ between the groups (P>0.05).

**Conclusion:**

This study showed that PAPPA levels are lower but free β-hCG levels are higher in single IVF versus normal pregnancies. This finding could be related to different placentation in intracytoplasmic sperm injection (ICSI) technique because of alterations in oocyte cytoplasm. Therefore, these markers may need to be adjusted in assisted re- productive technology (ART) conceptions. Further research should be done to obtain optimal cut-off for these markers in first trimester screening for detection of Down syndrome in ART pregnancies.

## Introduction

During gestation, pregnancy-associated plasma protein- A (PAPPA) is derivate from the placental trophoblasts and stromal cells at the placental-endometrial surface (1). The pregnancy-associated hormones such as human chorionic gonadotropin (hCG), progesterone and estradiol (E2) are present at high levels at the maternal-fetal surface during the remodeling period and thus could regulate trophoblast invasion (2). High levels of E2 in the placenta may lead to down regulation of the E2 receptors (3, 4). We hypothesized that a higher E2 levels on hCG injection days could result in a suboptimal placental-endometrial interface, and lead to reduced pregnancy-associated hormones concentrations.

Second trimester screening in pregnancy such as triple and quadruple tests are popular examinations used to evaluate for this hormonal imbalances; these examinations first one measure alpha-fetoprotein (AFP), unconjugated estriol, and β-hCG and then, inhibin-A. In case of abnormal second screening test results, complementary tests such as chorionic villus sampling and karyotyping should be performed (5, 6). Since the results of these tests may have negative psychological and emotional effects in pregnant women, the use of different methods to decrease false positive results was suggested (7).

*In vitro* fertilization (IVF) is an important treatment method for infertility. Some studies reported inaccuracy of screening tests in pregnancies developed by this method (8). The purpose of this study was to compare free β-hCG and PAPPA between single normal and IVF pregnancies at 12 gestational weeks.

## Materials and Methods

In this observational cohort study, 310 consecutive single IVF pregnancies and 720 single normal pregnancies attending for screenings of the first trimester, to tertiary health care centers, were enrolled at 11-13 week + 6 day gestational age. These women were recruited by random sampling. The study was approved by the Research Ethics Committee of Shahid Beheshti University of Medical Sciences, Tehran, Iran (013648749312). All eligible patients who were enrolled, signed an informed consent.

Inclusion criteria were single pregnancy and lack of complications (abortion, or ectopic or molar pregnancy). Also, subjects who did not have complete medical records, were excluded. Free β-hCG and PAPPA levels measured by kit (the BRAHMS free β-hCG and the PAPPA KRIPTOR CAL). This two automatic system based on immunofluorescence (mIU/mL) and were converted to multiple of the median (MOM) units, respectively and then compared between the groups (9). Also, other variables including maternal and paternal age, body mass index (BMI), gestational history, crown-rump length (CRL), and nuchal translucency (NT) on ultrasonography, were recorded.

t test was used to compare the results of the two groups. Chi-squared test was used for the analysis of differences in proportions among the groups. Data analysis was performed using SPSS software (SPSS, Chicago, Illinois, USA) version 24.0. P<0.05 was considered statistically significant.

## Results

A total of 1030 women were recruited in the present study; 30 women were excluded from the study due to various reasons, including absence of consent, or loss to follow up (data not shown). The mean age ± SD of the women in the IVF group and normal pregnancy group was 34.05 ± 4.728 and 33.44 ± 4.368 years, respectively (P=0.235).

Demographic characteristics such as BMI and reproductive histories including gravidity and parity and previous abortion did not vary significantly between the groups (P>0.05, Table 1). But, other confounders like fetus sex and maternal behaviors like smoking and alcoholism were not assessed.

**Table 1:** Demographic characteristics of enrolled patients


Group	IVF pregnancy n=300	Normal pregnancy n=700	P value

Maternal age (Y)	34.05 ± 4.728	33.44 ± 4.368	0.235
Paternal age (Y)	36.70 ± 4.739	36.15 ± 4.903	0.709
Gravidity (n)	1.23 ± 0.701	1.42 ± 0.744	0.101
Parity (n)	1.32 ± 0.478	1.10 ± 0.387	0.116
Living childs (n)	1.05 ± 0.229	1.09 ± 0.422	0.305
Prior abortions (n)	1.38 ± 0.976	1.32 ± 0.637	0.818
BMI (Kg/m^2^)	24.95 ± 3.169	24.54 ± 2.615	0.417


Data is shown as mean ± SD. Independent t test was used to compare different variables between the two groups. IVF; *In vitro* fertilization and BMI; Body mass index.

There was no significant difference in NT (1.54 ± 4.643 mm vs. 1.30 ± 1.004 mm respectively, P=0.235) and CRL (56.93 mm ± 7.552 vs. 57.55 mm ± 7.142 for IVF pregnancy and normal pregnant women, respectively, P=0.417) (Table 2).

**Table 2:** Laboratory screening and ultrasonography findings in two groups


Group	IVF pregnancyn=300	Normal pregnancyn=700	P value

PAPPA levels (mIU/mL)	3.75 ± 0.090	4.10 ± 2.251	0.026
β-hCG titrate(mIU/mL)	44.07 ± 31.366	37.28 ± 23.787	0.030
PAPPA (MOM)	1.19 ± 0.772	1.63 ± 4.375	0.167
β-hCG (MOM)	2.23 ± 2.367	1.67 ± 2.097	0.003
NT (mm)	1.54 ± 4.643	1.30 ± 1.004	0.235^a^
CRL (mm)	56.93 ± 7.552	57.55 ± 7.142	0.417^a^


Values are given as mean ± SD. ^a^; Independent t test was used to compare different variables between the two groups, IVF; *In vitro* fertilization, PAPPA; Pregnancy-associated plasma protein-A, β-hCG; Beta-human chorionic gonadotropin, NT; Nuchal translucency, and CRL; Crown-rump length.

Mean PAPPA levels was significantly lower (3.75 ± 0.090 vs. 4.10 ± 2.251 mIU/mL for IVF pregnancy and normal pregnant women, respectively, P=0.026) but β-hCG levels were significantly higher (44.07 ± 31.366 vs. 37.28 ± 23.787 mIU/mL for IVF pregnancy and normal pregnant women, respectively, P=0.030) in IVF pregnancies compared to normal pregnancies. After conversion of the data into MOM unit, no significant difference was found in PAPPA between the two groups (1.19 ± 0.772 vs. 1.63 ± 4.375 MOM for IVF pregnancy and normal pregnant women, respectively, P=0.167), however, a statistically significant difference was observed between the two groups in terms of β-hCG (1.54 ± 4.643 vs. 1.67 ± 2.097 MOM for IVF pregnancy and normal pregnant women, respectively, P=0.003) (Table 2).

## Discussion

Results of screening tests obtained for IVF pregnancies are equivocal and accurate risk assessment calculations for better interpretation is crucial. The purpose of this study was to compare free β-hCG and PAPPA levels between single normal and IVF pregnancies at about 12 gestational weeks. It was seen that PAPPA levels (as reported in mIU/mL) was lower but free β-hCG (as reported in both mIU/mL and MOM) was higher in IVF compared to normal pregnancies. In PAPPA levels reported in MOM, there was no significant difference between the two groups. The MOM values reported in the present research were adjusted for smoking status, maternal weight and ethnicity by laboratory software which could explain the differences in PAPPA results (9). Other factors like previous reproductive history, did not differ between the two groups.

This observational cohort study established significantly higher first-trimester β-hCG levels in intracytoplasmic sperm injection (ICSI) pregnancies compared to naturally conceived women (10). In contrast, free β-hCG titrate and NT thickness were similar between ART and normal pregnancies. Nevertheless, the majority of IVF/ICSI pregnant women had an elevated first-trimester combined screening risk estimation. Combined first-trimester screening (cFTS) combines the maternal age-related risk with the levels of maternal serum biomarkers such as free β-hCG, and PAPP-A, and fetal NT to determine the risk of trisomy 21, 18 and 13 (9).

Kagan et al. (8) found that IVF patients had up to 10% lower levels of PAPPA (MOM). Giorgetti et al. (10) reported that PAPPA levels were lower in IVF cases compared to normal pregnancy group but β-hCG levels were same between the groups. Also, they found no relationship between PAPPA levels and the etiology of infertility. However, in our study, significant differences were observed between the two groups for both PAPPA and β-hCG levels measured by ELISA (miL/ml). Giorgetti et al. (10) found that the PAPPA levels following intra uterine insemination (IUI) did not differ from natural conceptions, but in our research, the IUI-conceived women were not included.

Bellver et al. (11) reported lower PAPPA but higher β-hCG levels in IVF cases but no significant differences were found between the groups. However, both PAPPA and β-hCG levels were significantly different between the two groups in our study. Köşüş et al. (12) reported that the etiology of infertility affect β-hCG and PAPPA levels in IVF pregnancies and higher PAPPA level was seen in polycystic ovary (PCO) cases compared to male-factor infertile patients. However, infertility causes were not investigated in our research.

Orlandi et al. (13) reported that PAPPA level was significantly higher (up to 21 %) in IVF cases which was consistent with our data. Nevertheless, they evaluated singleton and twin pregnancy in a small population which was different from the population enrolled in the current study. Engels et al. (14) demonstrated that β-hCG after correction, was higher in IVF group compared to normal pregnancy group which was consistent with our results; however, PAPPA levels were lower in IVF/ICSI groups, they found that false positive rates were higher in IVF pregnant women and needed to be adjusted for better interpretation. However, unlike our study, their study was a retrospective study.

Gjerris et al. (15) reported similar β-hCG, but lower PAPPA levels and NT in ART groups compared to control; the inconsistency between their results and our findings may be due to the larger difference existed between the two groups included in their study compared to our study.

Cavoretto et al. (16) in a systemic review in 2017 reported that β-hCG was slightly higher in ICSI groups, but it did not vary significantly between IVF and normal pregnancy; however, PAPPA level was significantly lower in IVF group in which was consistent with our study. These authors suggested that such differences could be due to changes in the placentation of ART conceptions and recommended to define subgroups of ART conception to explore this discrepancy and better predict obstetrics outcomes. Cavoretto et al. (17) in a retrospective case-control study, found that PAPPA and CRL did not differ between IVF and normal pregnancy; however, β-hCG and NT were significantly higher in IVF group. They studied fresh and freeze single blastocyst transfer and found that pregnancy outcomes were similar when comparing the groups. These authors showed that this difference was not correlated with the pregnancy outcome but it could be due to changes or delay in embryogenesis or placentation and may depend on screening test performance. However, we did not perform blastocyst transfer and did not evaluate pregnancy outcomes. Our finding is in-line with the study as reported by Savasi et al. (18). They found that, free β-hCG levels are considerably greater in IVF/ICSI pregnancies. But, they studied another group of patients as oocyte donor IVF/ICSI cycles that could be different from our research that was done in autologous IVF cycles with their own eggs. They pointed that such variations could be due to the ART techniques.

A limitation of our study was lack of information on the causes of infertility, number and kind of embryos, freeze or fresh embryo transfer; also, we did not evaluate its correlation with biomarker levels such as PAPPA and free β-hCG. Moreover, we did not evaluate pregnancy outcomes in ART patients and controls.

One of the strengths of this cohort research is the large number of the participants. In addition, to avoid misinterpretation, we excluded the patients who received their ART treatment in another place.

PAPPA is an important indicator of the early growth and later development of the placenta (19). A prominent delay in fetal and placental growth leading to numerous metabolic disorders and greater risk of obstetrical complications (such as fetal growth restriction and preeclampsia). It is associated with assisted reproduction techniques and may also cause changes in serum markers levels (20, 21). Since just one corpus luteum is generally observed in pregnancies after freeze embryo transfer, which could explain the normal levels of PAPPA in these patients.

Finally, according to the results obtained in this study, it may be concluded that PAPPA levels are lower and free β-hCG levels are higher in single IVF compared to normal pregnancies. However, further studies are required to attain more definite results and assess other factors affecting first and second screening tests results.

## Conclusion

We found lower PAPPA but higher free β-hCG levels in single IVF compared to normal pregnancies. It could be related to different placentation in ICSI technique because of alteration in oocyte cytoplasm. Therefore, these markers may need to be adjusted in ART conceptions. Further research should be done to obtain optimal cut-off and more definite results for these biomarkers in first-trimester screening of Down syndrome in ART pregnancies.
